# Burden and Challenges in the Diagnosis and Treatment of Histoplasmosis Among Persons With Advanced HIV Disease in Uganda, 2025

**DOI:** 10.1093/ofid/ofag302

**Published:** 2026-05-14

**Authors:** Viola Kasone, Judith Kyokushaba, Patrick Semanda, Proscovia M Namuwenge, Henry Oundo, Sauda Namubiru, Grace Najjuka, Grace Nantege, Wilson Nyegenye, Rose Nakityo, Aida Namakula, Susan Nambozo, Bridget Ainembabazi, Joseph Kabanda, Julius Kalamya, Isaac Ssewanyana, Susan Nabadda, David Meya, Richard Kwizera

**Affiliations:** National Health Laboratory and Diagnostic Services, Ministry of Health, Luzira, Kampala, Uganda; National Health Laboratory and Diagnostic Services, Ministry of Health, Luzira, Kampala, Uganda; National Health Laboratory and Diagnostic Services, Ministry of Health, Luzira, Kampala, Uganda; STD/AIDS Control Program, Ministry of Health, Kampala, Uganda; National Health Laboratory and Diagnostic Services, Ministry of Health, Luzira, Kampala, Uganda; National Health Laboratory and Diagnostic Services, Ministry of Health, Luzira, Kampala, Uganda; National Health Laboratory and Diagnostic Services, Ministry of Health, Luzira, Kampala, Uganda; Division of Global HIV and TB, Global Health Centre, Centers for Disease Control and Prevention, US Embassy, Kampala, Uganda; National Health Laboratory and Diagnostic Services, Ministry of Health, Luzira, Kampala, Uganda; Division of Global HIV and TB, Global Health Centre, Centers for Disease Control and Prevention, US Embassy, Kampala, Uganda; National Health Laboratory and Diagnostic Services, Ministry of Health, Luzira, Kampala, Uganda; National Health Laboratory and Diagnostic Services, Ministry of Health, Luzira, Kampala, Uganda; STD/AIDS Control Program, Ministry of Health, Kampala, Uganda; Division of Global HIV and TB, Global Health Centre, Centers for Disease Control and Prevention, US Embassy, Kampala, Uganda; Division of Global HIV and TB, Global Health Centre, Centers for Disease Control and Prevention, US Embassy, Kampala, Uganda; National Health Laboratory and Diagnostic Services, Ministry of Health, Luzira, Kampala, Uganda; National Health Laboratory and Diagnostic Services, Ministry of Health, Luzira, Kampala, Uganda; Infectious Diseases Institute, College of Health Sciences, Makerere University, Kampala, Uganda; Infectious Diseases Institute, College of Health Sciences, Makerere University, Kampala, Uganda

**Keywords:** advanced HIV disease, antigenuria, galactomannan, histoplasmosis, Uganda

## Abstract

**Background:**

Data on the true burden of histoplasmosis in Uganda are limited, largely due to the scarcity of epidemiological studies. We aimed to describe the nationwide burden of histoplasmosis among persons with advanced HIV disease (AHD), highlighting the major challenges in diagnosis and treatment under routine HIV care.

**Methods:**

This was a prospective longitudinal study conducted at nine health facilities in Uganda. Persons with AHD (CD4 < 200 cells/mm^3^) were included and followed up for 2 years. Urine samples were sent to the Central Public Health Laboratories and tested using the *Histoplasma* galactomannan enzyme immunoassay in batches. Histoplasmosis was defined using standard criteria.

**Results:**

We enrolled 332 participants with AHD, and 66.6% were newly diagnosed with HIV. Over half were female (51.5%), with a median age of 35 years (interquartile range [IQR] = 29–42) for all participants. The median CD4 T-cell count was 73 cells/µL (IQR = 30.5–126.5). Eight (2.4%) participants were diagnosed with probable histoplasmosis. None of the cases received the recommended antifungals because they were not available in the country. After 2 years, 5 cases were still alive, 2 were lost to follow-up, and 1 died. Major challenges included missed diagnosis, low index of clinical suspicion, overlap of symptoms with those of pulmonary TB, high cost, limited access to antifungals such as voriconazole and itraconazole, radiology and laboratory investigations, and lack of knowledge about treatment guidelines.

**Conclusions:**

Histoplasmosis occurs at a low frequency among persons with AHD in Uganda. Numerous challenges hinder a definite diagnosis and treatment. However, despite these challenges, survival among persons with AHD in Uganda who have untreated histoplasmosis is relatively good.

Globally, over 100 000 cases of disseminated histoplasmosis have been estimated. In 2017, the World Health Organization (WHO) noted that disseminated histoplasmosis is a significant cause of mortality in AIDS patients [1, 2].

About 1.4 million people are estimated to be infected with HIV in Uganda, with over 17 000 AIDS-related deaths [3]. The prevalence of *Histoplasma* antigenuria among outpatient cohort with advanced HIV CD4 <100 cells/µL in Kampala, Uganda, was 2.5% [4]. Histoplasmosis is an infection caused by the dimorphic fungus *Histoplasma capsulatum*. The mode of infection in humans involves inhalation of fungal spores [5]. *Histoplasma capsulatum* thrives in environments with a lot of bird or bat droppings [6]. It is a soil dweller, and environmental exposure to spores is the major risk factor [7].

Uganda's geography and climate provide favorable conditions for the growth of *H. capsulatum*. Located on the equator, the country experiences a tropical climate with consistently warm temperatures (∼21–30 °C) and moderate-to-high rainfall, averaging 1000–1500 mm annually [8]. These warm and humid conditions support fungal survival in soil [9]. Uganda's landscape includes wetlands, forests, caves, lakes, and agricultural areas that sustain large bird and bat populations [10]. Accumulation of bird and bat droppings enriches the soil with organic matter, creating ideal conditions for *H. capsulatum* [11]. The most at-risk populations include persons exposed to a large inoculum, extremes of age, underlying emphysema, and immunocompromised patients [12].

Progressive disseminated histoplasmosis is commonly seen in persons with cell-mediated immunological defects like hematological malignancies, transplant recipients, prolonged use of corticosteroids, and HIV [13]. It is common in people living with HIV (PLHIV) due to the failure of cell-mediated immunity and is mostly seen in those with a CD4 cell count of <150 cells/µL [14]. Progressive disseminated histoplasmosis is therefore increasingly being recognized as a cause of morbidity in persons with advanced HIV disease (AHD) (CD4 < 200 cells/mm^3^) coming from endemic areas [1, 2]. About 20% of newly diagnosed PLHIV have AHD at diagnosis (preantiretroviral therapy [pre-ART]) in Uganda [15].

The true burden of HIV-associated disseminated histoplasmosis is unknown since it is not a notifiable disease [2]. Approximately half a million people globally get infected with histoplasmosis yearly; however, only about 100 000 people develop disseminated disease [16], with mortality rates ranging between 30% and 50% if treated and 100% if not [17]. A significant burden of disease is reported in South and Central America and Southeast Asia [18–20]. Histoplasmosis may be a neglected disease in Africa and has increased, mostly attributed to the growing HIV epidemic but is under-recognized [21]. There are limited epidemiological studies and data from East Africa, showing prevalence rates of histoplasmosis ranging from 1% to 9%, the majority of whom were clinically misdiagnosed as pulmonary TB or bacterial pneumonia among PLHIV with AHD, hospitalized febrile patients [22, 23].

In resource-constrained settings, the clinical presentations consistent with histoplasmosis are straightforward to identify, although they overlap with those of pulmonary TB. In AHD, pulmonary TB is most likely to be a comorbidity, which, when confirmed, can mask the undiagnosed histoplasmosis. If the pulmonary TB test is negative, then the patient may be managed as a community-acquired pneumonia unresponsive to antibiotics. Therefore, several cases of histoplasmosis in Uganda are probably misdiagnosed as smear-negative TB since Uganda is a TB endemic area, and there is a low index of clinical suspicion for histoplasmosis.

Culture is the gold standard for diagnosis of histoplasmosis. However, in the immunocompromised person, histoplasma polysaccharide antigen detection tests allow rapid diagnosis of disseminated histoplasmosis in urine, serum, BAL, and CSF samples before positive cultures can be identified. Antigen concentration is greatest in urine and can be used to monitor response to antifungal therapy and to identify relapsing patients [24]. However, cross-reactivity between the agents of other endemic mycoses should be considered in interpreting the results of the *Histoplasma* antigen tests. Point-of-care tests are available on the market and may be more useful in resource-constrained settings [25].

Data on the burden of histoplasmosis in Uganda are limited due to limited epidemiological studies, challenges in diagnosis, and treatment. In 1970, a study by Bezjak and Farsey found a 3.8% skin test sensitivity to *H. capsulatum* in Uganda [26]. In 2015, the estimation of the burden of all fungal infections in Uganda indicated that histoplasmosis occurred there, as evidenced by occasional case reports [27]. In 2016, a study by Bahr et al on seroprevalence of *H. capsulatum* among individuals with AHD in Kampala showed that 1.3% of subjects were serum *Histoplasma* IgG positive, indicating a past or resolved infection, and none were IgM positive, indicating the absence of a recent infection; antigen was not detected in any serum, urine, or CSF samples [23]. In 2020, a retrospective review of biopsy reports from Uganda over a 70-year period identified 64 cases of histoplasmosis (23 caused by *H. capsulatum* and 41 caused by *Histoplasma duboisii*), accounting for 9.2% of all deep fungal infections diagnosed by histology [28]. In 2023, a study on the prevalence of *Histoplasma* antigenuria using an enzyme immunoassay (EIA) in a cohort of outpatients with AHD in Kampala showed that 1.2% (4/388) were positive for *Histoplasma* antigen, and the clinical presentations of tuberculosis (TB) and histoplasmosis overlapped [4]. In 2024, the re-estimation of the burden of fungal infections in Uganda noted that the annual incidence of histoplasmosis was ∼646 per 100 000 PLHIV [29]. All these studies were mostly done in the country's capital city at the national referral hospital (NRH). Histoplasmosis diagnosis and treatment are not widely available in Uganda, although the national consolidated ART guidelines recommend it as a priority for patients with AHD, to be provided by the government of Uganda and its partners, PEPFAR and the Global Fund (https://hivpreventioncoalition.unaids.org/en/resources/consolidated-guidelines-prevention-and-treatment-hiv-and-aids-uganda-november-2022) [30].

In this study, we sought to describe the indicative national prevalence of histoplasmosis among persons with AHD in Uganda and to highlight the major challenges in diagnosing and managing this infection within routine HIV care, particularly in resource-constrained settings.

## METHODS

### Study Design and Setting

This was a prospective longitudinal study conducted from May 2023 to October 2025 at 9 health facilities in Uganda, including 1 NRH (Mulago), 7 regional referral hospitals (RRH; Mbarara, Soroti, Arua, Kabale, Mbale, Kayunga, and Hoima), and 1 Health Centre IV (Kisenyi HC IV). Active case identification was completed in 5 months, while follow-up took up to 2 years. The health facilities were selected because of the high volume of patients with AHD in care and the presence of laboratory enzyme-linked immunosorbent assay (ELISA) testing capacity. We used a laboratory reflex approach in which blood samples sent to the laboratory for routine CD4 testing among eligible patients as per the national guidelines that turned out to have a CD4 <200 cells/mm^3^ were actively flagged, and the study team was notified to obtain consent from the individual for study participation.

### Study Population and Inclusion Criteria

Persons with AHD at the study sites were considered. The only inclusion criteria were in- and outpatients with newly diagnosed PLHIV (not yet on ART) and all PLHIV (ART-experienced with nonsuppressed viral load) aged 5 years or more with confirmed AHD. AHD (CD4 < 200 cells/mm^3^) was diagnosed using only flow cytometry (BD FACSPresto™ analyzer) at the health facilities. We excluded patients with AHD who did not consent to participate in the study. Consecutive sampling was used to enroll participants who fit the inclusion criteria. The sample size was calculated using Cochran's (1977) formula [31] ($n_{0}=(Z^{2}p(1-p))/e^{2}$) for large or unknown populations, ensuring a 95% confidence level (*Z* = 1.96), a 5% margin of error (*e* = 0.05), and a prevalence of 24.3% [32] (*P* = .243), resulting in a standard minimum sample size of 283. Assuming a 10% nonresponse, the adjusted minimum sample size was 314.

### Study Procedures

People with HIV were routinely screened for AHD. Those who fit the study inclusion criteria were consented to participate in it. As per standard of care, urine samples were collected to test for TB lipoarabinomannan from eligible study participants (PLHIV with CD4 <200 cells/mm^3^). The remnant urine samples were used for *Histoplasma* Galactomannan EIA (IMMY™; Norman, OK, United States) testing following the manufacturer's instructions. Urine samples were sent to the Central Public Health Laboratories (CPHL) for testing using the *Histoplasma* Galactomannan EIA and were tested in batches. Other clinical, laboratory, radiology, and outcome data were obtained from the patients’ hospital records; all of these were part of routine care and not part of the study enrollment. Treating clinicians received the Histoplasma antigen results. However, culture and histopathology were not available to make a definite diagnosis for histoplasmosis as per the standard case definition. In addition, antifungal therapies, apart from amphotericin B, the recommended itraconazole and voriconazole, were not available at the facilities during the study period. The patients were followed up as part of routine HIV care in the respective health facilities for 2 years, and the data were collected by reviewing hospital and clinic records.

The primary outcome of interest was histoplasmosis, which we defined using criteria adapted from the European Organization for Research and Treatment of Cancer and the Mycoses Study Group Education and Research Consortium criteria (EORTC/MSGERC) [33, 34] (Table 1). None of the probable histoplasmosis cases received antifungal treatment (itraconazole and voriconazole) because they were not available. However, all cases received treatment for AHD and other comorbidities. Antiretroviral therapy (ART) was initiated after diagnosing the AHD and histoplasmosis at baseline following the WHO test and treat guidelines.

**Table 1.** Case Definition for *Histoplasmosis* Infections

Classification	Proven/Definite	Probable	Possible
Definition	Histopathologic, cytopathologic, or direct examination of specimen obtained by needle aspiration or biopsy consistent with histoplasmosis	Clinical syndrome congruent with histoplasmosis	Clinical syndrome congruent with histoplasmosis
		AND	AND
		Presence of predisposing condition	Presence of predisposing condition with at least one of the following:
	OR	AND	>4 times rise in complement fixation titers taken at least 2 weeks apart Complement fixation ≥32 Immunodiffusion assay showing H band Immunodiffusion assay showing M band after prior negative immunodiffusion assay
	Culture obtained by sterile procedure from a site suspected of an infectious disease process consistent with histoplasmosis	Positive antigen test	

### Statistical Analysis

Data were analyzed using SAS 9.4 (SAS Institute, Cary, NC, United States). We used descriptive methods, frequencies, proportions, and summary statistics to describe the characteristics of the study population. We estimated prevalence with exact 95% binomial confidence intervals (CIs) overall and by site; given the small number of histoplasmosis cases, no formal hypothesis testing was performed.

### Ethical Consideration and Consent to Participate

Informed consent was obtained from all subjects involved in the study. The study was conducted in accordance with the Declaration of Helsinki and approved by the Research Ethics Committee of the Uganda National Health Laboratory Services (protocol number UNHL-2023-58; date of approval: 3 February 2023) and the Uganda National Council for Science and Technology (HS2803ES). This study was also reviewed and approved by the CDC Institutional Review Board.

## RESULTS

### Characteristics of the Study Population

We enrolled 332 persons with AHD from 9 health facilities, which represented 20% (332/1660) of PLHIV who were eligible for CD4 testing at the facilities during the study time, among which 66.6% were newly diagnosed with HIV. Over half were female (51.5%) with an overall median age of all participants of 35 years (interquartile range [IQR] = 29–42). The median CD4 cell count was 73 cells/µL (IQR = 30.5–126.5), and the majority of participants (82.5%) were outpatients (Table 2).

**Table 2.** Location, Time, and Demographic Characteristics of Study Participants

Characteristic	Median (IQR) or N (%) (N = 332)
**Age (y),** median (IQR)	35 (29–42)
**Age category,** n (%)	
5–24	45 (13.6)
25–34	112 (33.7)
35–44	108 (32.5)
45+	65 (19.6)
Missing	2 (0.6)
**Sex, n (%)**	
Female	171 (51.5)
Male	160 (48.2)
Missing	1 (0.3)
**Care level**	
Inpatient	56 (16.9)
Outpatient	274 (82.5)
Missing	2 (0.6)
**ART duration at presentation categories, n (%)**	
<1 m	120 (36.1)
1–6 m	109 (32.9)
>6 m	92 (27.7)
Missing	11 (3.3)
**CD4 (cells/µL), median (IQR)**	73 (31–127)
**CD4 categories, n (%)**	
100–199	123 (37.1)
0–99	193 (58.1)
<50 (below the lower detection limit)^a^	16 (4.8)
**Patient category, n (%)**	
New PLHIV	221 (66.6)
Nonsuppressed >1000 copies/mL	37 (11.1)
Returning to care after LTU	44 (13.2)
WHO stage ¾	24 (7.3)
Missing	6 (1.8)
**Other clinical symptoms, n (%)**	
Cough and ≥1 constitutional symptom	105 (34.5)
≥1 constitutional symptom (without cough)	33 (10.9)
Skin condition (without any other symptom)	24 (7.9)
Skin condition with and other symptom (cough and weight loss)	15 (4.9)
Fever (without any other symptom)	13 (10.9)
Cough (without any other symptom)	12 (4.0)
Chest pain and other symptom (cough, fever, weight loss, or others)	5 (1.6)
Others	36 (11.8)
None	51 (16.8)
Missing	28
**Other comorbidities, n (%)**	
Cryptococcal antigenemia	31 (9.3)
Aspergillus GM antigenemia	119 (36)
Tuberculosis^b^	96 (28.9)
Pneumonia^c^	24 (7.2)

Abbreviations: GM, galactomannan; IQR, interquartile range; LTFU, lost to follow-up; PLHIV, people living with HIV; WHO, World Health Organization.

^a^The 16 missing CD4 counts were a result of the CD4 machines’ limitations. These machines report patients with extremely low CD4 cell counts as “<50” for example, when the absolute cell count is lower than the machines’ lower detection limit. Based on the machines’ performance and validation, the indeterminate results were in the range of 0–99 cells/µL. This means that even though the exact value was not generated, the patients were still included in the analysis since they were above the AHD threshold.

^b^Tuberculosis—participants with either a positive urine TB LAM and/or a positive GeneXpert result were considered to have tuberculosis.

^c^Pneumonia was defined as the presence of lung parenchymal infiltrates or consolidation on chest radiograph (X-ray), consistent with an acute lower respiratory tract infection.

### Summary of Histoplasmosis Cases in AHD

#### Prevalence of Histoplasmosis

Among the 332 participants tested, 8 (2.4%; 95% CI: 1.0%–4.8%) were positive for *Histoplasma* galactomannan antigen in urine. Positivity varied across the sites (Table 3). As per the EORTC/MSGERC case definition, all 8 cases were diagnosed with probable histoplasmosis. There were 4 males and 4 females, with ages ranging between 28 and 52 years. Their occupations included peasant farmer, driver, police officer, car washing bay attendant, and canteen attendant.

**Table 3.** Distribution of Positivity for Histoplasmosis by Site

Region	Facility	Level	Number of Participants	Number Positives for Histoplasmosis	Percentage Positivity
Central	Mulago Hospital	NRH	34	1	2.9
Central	Kayunga Hospital	RRH	48	2	4.1
Central	Hoima Hospital	RRH	12	0	0
Central	Kisenyi Hospital	HC IV	40	0	0
Western	Mbarara Hospital	RRH	62	1	1.6
Western	Kabale Hospital	RRH	24	0	0
Eastern	Soroti Hospital	RRH	46	1	2.2
Eastern	Mbale Hospital	RRH	13	1	7.7
Northern	Arua Hospital	RRH	53	2	3.8
	**Overall**		**332**	**8**	**2.4**

NRH, National Referral Hospital; RRH, Regional Referral Hospital; HC IV, Health Centre IV.

#### Predisposing Factors and Other Comorbidities

All 8 cases had AHD as the major predisposing factor with a CD4 cell count of 13–130 cells/µL. Only 2 participants had a suppressed HIV viral load. Other comorbidities included active or prior pulmonary TB, *Aspergillus* GM antigenemia, oral candidiasis, cryptococcosis, severe malaria, sepsis, and hepatitis B.

#### Clinical Presentation

Clinical symptoms congruent with histoplasmosis included fever, weight loss, skin rash, cough, diarrhea, abdominal pain, chest pain, dyspnea, hepatosplenomegaly, headache, joint pains, constipation, and chills. Other clinical symptoms included body weakness, wasting, mild pallor, dehydration, night sweats, mild anemia, oral sores, ear pain, hematuria, jaundice, oral thrush, and whitish vaginal discharge.

#### Final Diagnosis

All 8 cases had confirmed AHD and probable histoplasmosis. Other confirmed diagnoses included active pulmonary TB (n = 3), post-TB lung disease (n = 2), oral candidiasis (n = 3), cryptococcal antigenemia (n = 2), cryptococcal meningitis (n = 1), severe malaria (n = 1), sepsis (n = 1), hepatitis B (n = 1), and cervical cancer (n = 1).

#### Treatment

None of the 8 cases received treatment for probable histoplasmosis. Whereas the treating clinicians received the histoplasma antigen results, additional confirmatory case-definition investigations for definite histoplasmosis were not available. In addition, recommended antifungal therapies, including itraconazole and voriconazole, were not available at the facilities during the study period. However, all cases received ART with/without cotrimoxazole to manage the AHD, and they continued ART over time. Those with active TB were managed with anti-TB (2-month intensive phase with rifampicin (R), isoniazid (H), pyrazinamide (Z), and ethambutol (E), followed by a 4-month continuation phase of rifampicin and isoniazid (RH) = 2RHZE + 4RH) with/without pyridoxine. Oral candidiasis and cryptococcal antigenemia were managed with oral fluconazole. Cryptococcal meningitis was managed with ambisome, fluconazole, flucytosine, and potassium chloride. Sepsis was managed with IV antibiotics, and severe malaria was managed with antimalarials. There was no documentation for the treatment of post-TB lung disease, hepatitis B, and cervical cancer.

#### Clinical Outcomes

Five cases with probable histoplasmosis were still alive after 2 years, 1 died after only 2 weeks on ART and 2 were lost to follow-up at the review time (one for about a year and the other for >90 days; Table 4).

**Table 4.** Summary of Histoplasmosis Cases Among Patients With AHD in Uganda

Case No.	Sex/Age	Occupation	Predisposing Condition Timing of AHD Diagnosis	Other Underlying Conditions	Clinical Syndrome Congruent With Histoplasmosis	Other Symptoms	Urine *Histoplasma* GM EIA	Other Laboratory Investigations	Final Diagnosis	Treatment	Outcome
1	M/47	Peasant farmer	Outpatient, newly diagnosed PLHIV	None	Fever, weight loss, skin rash, cough, diarrhea, abdominal pain	Body weakness, sasted, mild pallor, moderate dehydration	Positive	CD4: 15	AHD Probable histoplasmosis	ART (TDF/3TC/DTG) Cotrimoxazole	LTFU
2	M/29	Driver	Outpatient, newly diagnosed PLHIV	Pulmonary TB, oral candidiasis	Fever, weight loss, cough, chest pain, dyspnea, hepatosplenomegaly	Night sweats, wasted, body weakness, oral candidiasis	Positive	CD4: 13 Asp GM: POS Urine LAM: POS MTB/RIF: POS	AHD Probable histoplasmosis Pulmonary TB Oral candidiasis	ART (TDF/3TC/DTG) Cotrimoxazole Anti-TB (2RHZE + 4RH) Pyridoxine Fluconazole	Alive
3	M/52	Police officer	Outpatient, VL nonsuppressed PLHIV	History of pulmonary TB	Mild weight loss	Mild anemia	Positive	CD4: 98	AHD Probable histoplasmosis Post-TB lung disease	ART (TDF/3TC/DTG) Cotrimoxazole	LTFU
4	F/28	Peasant farmer	Outpatient, VL nonsuppressed PLHIV	Cryptococcal antigenemia	Skin rash, fever, lower abdominal pain, headache, joint pains	None	Positive	CD4: 32 sCrAg: POS Urine LAM: POS	AHD Probable histoplasmosis Cryptococcal antigenemia	ART (TDF/3TC/EFV) Fluconazole anti-TB (2RHZE + 4RH)	Died
5	F/33	Peasant farmer	Outpatient, newly diagnosed PLHIV	Cryptococcal antigenemia, oral candidiasis, severe malaria, sepsis	Skin rash, fever, weight loss, hepatosplenomegaly	Oral sores, ear pain, body weakness, wasted, dehydrated, oral candidiasis	Positive	CD4: 140 Asp GM: POS sCrAg: POS	AHD Probable histoplasmosis Cryptococcal antigenemia Oral candidiasis Severe malaria Sepsis	ART (TDF/3TC/DTG) IV Ampiclox Fluconazole Antimalarials	Alive
6	M/42	Car washing bay attendant	Outpatient, newly diagnosed PLHIV	Pulmonary TB	Weight loss, constipation, chills	Night sweats, hematuria	Positive	CD4: 130 Urine LAM: POS	AHD Probable histoplasmosis Pulmonary TB	ART (TDF/3TC/EFV) Cotrimoxazole Anti-TB (2RHZE + 4RH) TPT (3HP)	Alive
7	F/39	Canteen attendant	Inpatient, newly diagnosed PLHIV	Pulmonary TB, cryptococcal meningitis, oral candidiasis	Fever, cough, weight loss, headache	Night sweats, wasted, mild pallor, jaundice, body weakness, oral thrush	Positive	CD4: 150 VL: nonsuppressed CSF CrAg: POS	AHD Probable histoplasmosis Pulmonary TB Cryptococcal meningitis Oral candidiasis	ART (TDF/3TC/EFV) Cotrimoxazole Anti-TB (2RHZE + 4RH) Pyridoxine Ambisome Fluconazole Flucytosine KCL	Alive
8	F/36	Peasant farmer	Inpatient, VL nonsuppressed PLHIV	History of pulmonary TB, hepatitis, cervical cancer	Cough, lower abdominal pain, headache	Whitish vaginal discharge	Positive	CD4: 92 HBV: POS HPV: POS AST: 117.1 U/L ALT: 104.4 U/L ALP: 101.5 U/L	AHD Probable histoplasmosis Post-TB lung disease Hepatitis B Cervical cancer	ART (TDF/3TC/DTG)	Alive

Abbreviations: AHD, advanced HIV disease; M, male; F, female; TDF/3TC/DTG, tenofovir/lamivudine/dolutegravir; TDF/3TC/EFV, tenofovir/lamivudine/efavirenz; TB, tuberculosis; Asp GM, aspergillus galactomannan antigen; sCrAg, serum cryptococcal antigen; LAM, lipoarabinomannan; MTB/RIF, mycobacteria/rifampicin resistance; HBV, hepatitis B virus surface antigen; HPV, human papillomavirus; POS, positive; NEG, negative; ART, antiretroviral therapy; 2RHZE + 4RH, rifampicin, isoniazid, pyrazinamide, and ethambutol + rifampicin and isoniazid; IV, intravenous; TPT, Tuberculosis Preventive Treatment; 3HP, rifapentine and isoniazid; KCL, potassium chloride; LTFU, loss to follow-up; CD4, cluster of differentiation 4; VL, viral load.

## DISCUSSION

### Burden of Histoplasmosis in AHD in Uganda

This study demonstrated that histoplasmosis occurs at a low frequency (2.4%) among persons with AHD in Uganda. This agrees with previous studies done in Uganda [4, 23, 26–29]. There was an equal distribution of male versus female among the 8 cases identified, all of whom were young adults under 55 years of age. Occupational exposure to histoplasmosis is common. High-risk jobs include construction, demolition, agriculture, landscaping, and work involving soil disturbance or cleaning in caves or bird/bat-infested structures [35]. Half of our cases were peasant farmers, while the other half were not in “soil-disturbing” occupations, demonstrating how histoplasmosis can affect PLHIV with AHD even in the absence of high-risk occupations.

Advanced HIV disease and other HIV-related comorbidities were the major risk factors for our cases. One participant had cervical cancer, although there was no documentation of her treatment for the cervical cancer. However, other known risk factors include age over 65 years, active cancer with immune-compromising treatments, immune compromise due to medications or other medical conditions, and diabetes mellitus [1]. Due to the immunocompromised nature of these cases with AHD, it was not a surprise to us that the other commonly registered opportunistic infections (TB, cryptococcosis, and candidiasis) occurred in the same people. Only one case had no other underlying conditions except AHD.

The clinical manifestations seen in our participants were consistent with the wide spectrum of histoplasmosis manifestations in AHD [36]. Disseminated histoplasmosis often resembles and can be misdiagnosed as pulmonary TB [37]. It should therefore be ruled out among patients with presumptive TB. The presence of skin lesions, which can be biopsied, is helpful if present. Gastrointestinal symptoms are often prominent in disseminated histoplasmosis, unlike in TB [38]. Pancytopenia is more profound than in other patients with AHD [39]. However, we did not have a record of full blood counts to check for Pancytopenia. Among our cases, 3 had skin lesions, while 4 had gastrointestinal symptoms suggestive of disseminated disease. However, histopathology and culture were not available for confirmation.

In this study, mortality was ∼13% (1/8). Previous studies have demonstrated mortality rates of 30%–50% if treated and 100% if not [17]. The low mortality observed in our study could partially be explained by the fact that patients initiated ART, which could have restored their immunity. Outcomes were assessed through routine clinical follow-up for up to 2 years after enrollment. Mortality and clinical status were extracted from medical records at the last documented follow-up visit. Therefore, outcomes represent cumulative status over the follow-up period rather than a fixed time point such as 2-week or 1-year mortality.

### Challenges in the Diagnosis of Histoplasmosis

Even with a clinical suspicion, the major challenge in confirming the diagnosis is due to poor access to pathology, culture, radiology, and definitive laboratory investigations. This study was carried out under routine AHD care in a resource-constrained setting using the available resources. Therefore, we could not confirm any proven/definite histoplasmosis cases due to a lack of histopathologic and culture evidence. These tests are not routinely performed at the regional and national-level health facilities in Uganda. Patients would need to incur the costs of these tests from private facilities. These facilities also lack trained pathologists and mycologists to make a definite diagnosis.

We did not have a record of full blood counts to check for pancytopenia. Full blood counts are done in this population when anemia or sepsis is suspected. Radiology documentation was also missing for all 8 cases identified. Radiology is routinely done for patients with TB-like syndrome. Even in such cases, the patient incurs the cost of a chest X-ray or computerized tomography scan. Most of the chest radiographs may be normal, and 50% will show bilateral infiltrates or nodular opacities [40]. Any organ in the body can be affected, but it is mostly seen in the lung, liver, spleen, brain, and adrenals. Hepatomegaly and splenomegaly are very common in this disease. Only one case had liver function tests done due to signs of hepatitis B. It was unclear whether these tests were not done or poorly documented.

The *Histoplasma* Galactomannan EIA (IMMY™) test used in this study measures the presence of the polysaccharide antigen in the blood. It was previously found to be cross-reactive with only *Paracoccidioides*, *Blastomyces*, and some *Candida* specimens [41]. Positive tests should be confirmed in areas or patient groups where these organisms are endemic or a risk. Coinfection with other fungal pathogens was present in some of our patients, including cryptococcosis (n = 3/8), oral candidiasis (n = 3/8), and *Aspergillus* galactomannan positivity (n = 1/8). Only 3 of the participants had oral candidiasis with a potential for cross-reactivity. The cryptococcal antigen test is based on the detection of the capsular polysaccharide of *Cryptococcus neoformans*, glucuronoxylomannan, which is distinct from the *Histoplasma* galactomannan molecule; assay cross-reaction is not likely to occur. There may be cross-reaction between the Histoplasma and Aspergillus galactomannan assays, as they share the common galactomannan molecule, although this has not been documented before. It was not possible to exclude co-infection or assay cross-reaction without culture or histopathology, which is not always feasible in resource-constrained settings and was a limitation of this study. The antigen detection used to detect other opportunistic infections like TB and HBV is immunologically unrelated to fungal polysaccharide antigens, and so cross-reactivity with *Histoplasma* galactomannan is biologically implausible.

The *Histoplasma* Galactomannan EIA (HGM201) antigen test used in this study is also not routinely available. This test was acquired for this study, and it costs about 6 USD per test [42]. Urine samples were sent to the CPHL and tested in batches, which greatly affected the turnaround time of results. Each ELISA plate run requires positive and negative controls; running only a few samples wastes reagents and controls, increasing the cost per test. Point-of-care urine tests are also not available, which could provide affordable and quick alternatives.

### Challenges in the Treatment of Histoplasmosis

Treatment guidelines for histoplasmosis among PLHIV recommend the use of amphotericin B, itraconazole, voriconazole, and ART [43]. In our study, done under routine HIV care, none of the 8 cases received antifungal treatment for histoplasmosis. Our cases only received the standard of care for AHD and other opportunistic infections identified. There is probably a lack of knowledge about the treatment guidelines for histoplasmosis among the health workers. Liposomal amphotericin B is available as an essential drug at Mulago and Kiruddu NRHs only, mostly used to manage HIV-related cryptococcal meningitis in Uganda. However, other facilities do not have access, so refer patients to these 2 NRHs. Itraconazole and voriconazole are not available on the Ugandan essential drugs list. So, patients must buy costly generic brands from private pharmacies. However, despite these challenges, we registered a 63% (5/8) survival rate of untreated probable histoplasmosis among patients with AHD in Uganda. Survival outcomes presented in Table 4 were at 24 months after enrollment, except for the death that occurred 2 weeks after ART initiation. The 8 patients were initiated on ART at baseline as per the WHO “test and treat” guidelines and remained on ART unless they were lost to follow-up or deceased. From the clinical documentation, the likely causes of death were complications of AHD with cryptococcosis and/or TB, rather than histoplasmosis. It is also likely that the high survival rate could have been possible due to the treatment with liposomal amphotericin B for the 1 patient who was co-infected with cryptococcal meningitis.

### Limitations of the Study

The major limitation of this study was the lack of some laboratory investigations, such as full blood count, liver function tests, histopathology, fungal culture, and radiology, to guide a proper case definition for a definite diagnosis. The lack of confirmatory testing means all cases are probable, which risks over- or underestimation of the actual burden. None of the cases was directly managed for probable histoplasmosis, which could have improved the observed survival rate. Histoplasma antigen-positive cases may be false positives due to cross-reactivity with *Candida* in some cases, which may overestimate the incidence. The findings are based on data from only 9 facilities, and this may not be fully representative of the national picture.

## CONCLUSIONS

Our study offers the first national signal of probable Histoplasmosis burden among persons with AHD in Uganda, suggesting that the infection occurs at a low frequency among this population. Numerous challenges exist, hindering proper diagnosis and treatment. However, despite these challenges, survival of untreated histoplasmosis is relatively good among patients with AHD in Uganda. This study adds data points to better understand the global epidemiology of histoplasmosis.

### Suggested Next Steps

Incorporating point-of-care urine antigen testing into the AHD package of care may be helpful to facilitate routine screening for histoplasmosis in this high-risk population. Such integration would enhance early detection and improve clinical management, particularly in settings where diagnostic capacity remains limited. Itraconazole and voriconazole may be considered for Uganda's essential medicines list to ensure the effective management of confirmed histoplasmosis cases, particularly within AHD care, where timely access to appropriate antifungal treatment is critical. It is necessary for health workers at national, RRHs, and district health facilities to be trained in the diagnosis and treatment of histoplasmosis. Enhanced laboratory capacity, improved documentation at the health facilities, and routine follow-up of cases could improve patient clinical outcomes. Expanding surveillance to more regions and facility levels, especially among the patients with AHD would strengthen future estimates and help guide more comprehensive national responses.

## References

[1] Bongomin F, Kwizera R, Denning DW. Getting histoplasmosis on the map of international recommendations for patients with advanced HIV disease. J Fungi (Basel) 2019; 5:80. 31480775 10.3390/jof5030080 PMC6787619

[2] Bongomin F, Gago S, Oladele RO, Denning DW. Global and multi-national prevalence of fungal diseases—estimate precision. J Fungi 2017; 3:57. 10.3390/jof3040057 PMC5753159 29371573

[3] Uganda AIDS Commission. Uganda HIV & AIDS Factsheet: Uganda AIDS Commission; 2023. Available at: https://www.uac.go.ug/media/attachments/2024/01/23/hiv-aids-factsheet-2023.pdf

[4] Sekar P, Nalintya E, Kwizera R, et al Prevalence of histoplasma antigenuria among outpatient cohort with advanced HIV in Kampala, Uganda. J Fungi 2023; 9:757. 10.3390/jof9070757 PMC10381727 37504745

[5] Bahr NC, Antinori S, Wheat LJ, Sarosi GA. Histoplasmosis infections worldwide: thinking outside of the Ohio river valley. Curr Trop Med Rep 2015; 2:70–80. 26279969 10.1007/s40475-015-0044-0 PMC4535725

[6] Diaz JH . Environmental and wilderness-related risk factors for histoplasmosis: more than bats in caves. Wilderness Environ Med 2018; 29:531–40. 30266238 10.1016/j.wem.2018.06.008

[7] Zeidberg L, Ajello L. Environmental factors influencing the occurrence of *Histoplasma capsulatum* and *Microsporum gypseum* in soil. J Bacteriol 1954; 68:156–9. 13183922 10.1128/jb.68.2.156-159.1954 PMC357359

[8] The Climate of Uganda: Blue Green Atlas; [05 February 2026]. Available at: https://bluegreenatlas.com/climate/uganda_climate.html#:∼:text=In%20summary:,1%2C600%20mm%20(64%20in)

[9] Yiallouris A, Pana ZD, Marangos G, et al Fungal diversity in the soil mycobiome: implications for ONE health. One Health 2024; 18:100720. 38699438 10.1016/j.onehlt.2024.100720 PMC11064618

[10] Okia R . Bird conservation in Uganda: Avian Conservation Uganda; 2023. Available at: https://acugs.org/tag/bird-conservation-in-uganda/#:∼:text=Protected%20areas%20like%20Queen%20Elizabeth,BIODIVERSITY%20AND%20ECOSYESTEM%20SERVICES

[11] de Perio MA, Benedict K, Williams SL, et al Occupational histoplasmosis: epidemiology and prevention measures. J Fungi (Basel) 2021; 7. 10.3390/jof7070510 PMC8306883 34206791

[12] Efrim ND, Dumea E, Cernat RC. Epidemiology of histoplasmosis. In: Histoplasmosis—a comprehensive study of epidemiology, pathogenesis, diagnosis, and treatment. IntechOpen, 2023.

[13] Kauffman CA . Histoplasmosis: a clinical and laboratory update. Clin Microbiol Rev 2007; 20:115–32. 17223625 10.1128/CMR.00027-06 PMC1797635

[14] Cernat R-C . HIV-associated histoplasmosis. In: Histoplasmosis—a comprehensive study of epidemiology, pathogenesis, diagnosis, and treatment: IntechOpen, 2023.

[15] Namuwenge P, Watiti S. Advanced HIV Disease Update: Uganda: Uganda Ministry of Health; 2020. Available at: https://cquin.icap.columbia.edu/wp-content/uploads/2020/07/4.-Uganda-AHD-Meeting_Country-Template.pdf

[16] Denning DW . Minimizing fungal disease deaths will allow the UNAIDS target of reducing annual AIDS deaths below 500 000 by 2020 to be realized. Philos Trans R Soc B Biol Sci 2016; 371:20150468. 10.1098/rstb.2015.0468 PMC5095544 28080991

[17] Brown GD, Denning DW, Gow NA, Levitz SM, Netea MG, White TC. Hidden killers: human fungal infections. Sci Transl Med 2012; 4:165rv13–rv13. 10.1126/scitranslmed.3004404 23253612

[18] Nacher M, Leitao TS, Gómez BL, et al The fight against HIV-associated disseminated histoplasmosis in the Americas: unfolding the different stories of four centers. J Fungi 2019; 5:51. 10.3390/jof5020051 PMC6617033 31212897

[19] Adenis AA, Valdes A, Cropet C, et al Burden of HIV-associated histoplasmosis compared with tuberculosis in Latin America: a modelling study. Lancet Infect Dis 2018; 18:1150–9. 30146320 10.1016/S1473-3099(18)30354-2 PMC6746313

[20] Baker J, Setianingrum F, Wahyuningsih R, Denning DW. Mapping histoplasmosis in South East Asia—implications for diagnosis in AIDS. Emerg Microbes Infect 2019; 8:1139–45. 31364950 10.1080/22221751.2019.1644539 PMC6711083

[21] Oladele RO, Ayanlowo OO, Richardson MD, Denning DW. Histoplasmosis in Africa: an emerging or a neglected disease? PLoS Negl Trop Dis 2018; 12:e0006046. 29346384 10.1371/journal.pntd.0006046 PMC5773084

[22] Lofgren SM, Kirsch EJ, Maro VP, et al Histoplasmosis among hospitalized febrile patients in northern Tanzania. Trans R Soc Trop Med Hyg 2012; 106:504–7. 22742942 10.1016/j.trstmh.2012.05.009 PMC3392419

[23] Bahr NC, Sarosi GA, Meya DB, et al Seroprevalence of histoplasmosis in Kampala, Uganda. Sabouraudia 2016; 54:295–300. 10.1093/mmy/myv081 PMC4837699 26527637

[24] Hanzlicek A, Meinkoth J, Renschler J, Goad C, Wheat L. Antigen concentrations as an indicator of clinical remission and disease relapse in cats with histoplasmosis. J Vet Intern Med 2016; 30:1065–73. 27158815 10.1111/jvim.13962 PMC5084835

[25] Kuate MPN, Abessolo Abessolo H, Denning DW, Stone NR, Ndip RN. Diagnosing disseminated histoplasmosis in advanced HIV/AIDS disease in Cameroon using a point of care lateral flow assay. Ther Adv Infect Dis 2022; 9:20499361221132133. 36277298 10.1177/20499361221132133 PMC9583206

[26] Bezjak V, Farsey SJ. Prevalence of skin sensitivity to histoplasmin and coccidioidin in varous Ugandan populations. Am J Trop Med Hyg 1970; 19:664–9. 5425506 10.4269/ajtmh.1970.19.664

[27] Parkes-Ratanshi R, Achan B, Kwizera R, Kambugu A, Meya D, Denning DW. Cryptococcal disease and the burden of other fungal diseases in Uganda; where are the knowledge gaps and how can we fill them? Mycoses 2015; 58(S5):85–93. 26449512 10.1111/myc.12387

[28] Kwizera R, Bongomin F, Lukande R. Deep fungal infections diagnosed by histology in Uganda: a 70-year retrospective study. Med Mycol 2020; 58:1044–52. 32242631 10.1093/mmy/myaa018 PMC7657094

[29] Bongomin F, Kwizera R, Namusobya M, et al Re-estimation of the burden of serious fungal diseases in Uganda. Ther Adv Infect Dis 2024; 11:20499361241228345. 38328511 10.1177/20499361241228345 PMC10848809

[30] Available at: https://hivpreventioncoalition.unaids.org/en/resources/consolidated-guidelines-prevention-and-treatment-hiv-and-aids-uganda-november-2022

[31] Cochran WG , Sampling techniques. 3rd ed. New York: John Wiley & Sons, 1977.

[32] Sturny-Leclère A, Françoise U, Badjé AD, et al Histoplasma antigenuria prevalence in patients with advanced HIV disease in Côte d'Ivoire: a prospective trial ancillary study. Clin Infect Dis 2025; 81:950–8. 40377456 10.1093/cid/ciaf237

[33] De Pauw B, Walsh TJ, Donnelly JP, et al Revised definitions of invasive fungal disease from the European Organization for Research and Treatment of Cancer/Invasive Fungal Infections Cooperative Group and the National Institute of Allergy and Infectious Diseases Mycoses Study Group (EORTC/MSG) consensus group. Clin Infect Dis 2008; 46:1813–21. 18462102 10.1086/588660 PMC2671227

[34] Donnelly JP, Chen SC, Kauffman CA, et al Revision and update of the consensus definitions of invasive fungal disease from the European Organization for Research and Treatment of Cancer and the Mycoses Study Group Education and Research Consortium. Clin Infect Dis 2020; 71:1367–76. 31802125 10.1093/cid/ciz1008 PMC7486838

[35] CDC. About Histoplasmosis and Work 2024 [12 Sept 2025]. Available at: https://www.cdc.gov/niosh/histoplasmosis/about/index.html

[36] Rahim MA, Zaman S, Amin MR, Uddin KN, Chowdhury J. Histoplasmosis: an emerging or neglected disease in Bangladesh? A systematic review. Oman Med J 2020; 35:e91. 32095275 10.5001/omj.2020.09 PMC7024808

[37] Anot K, Sharma S, Gupta M, Kaur D. Disseminated histoplasmosis and tuberculosis: dual infection in a non-endemic region. BMJ Case Rep CP 2020; 13:e235531. 10.1136/bcr-2020-235531 PMC7449591 32843417

[38] Ekeng BE, Itam-Eyo AE, Osaigbovo II, et al Gastrointestinal histoplasmosis: a descriptive review, 2001–2021. Life 2023; 13:689. 36983844 10.3390/life13030689 PMC10051669

[39] Ekeng BE, Elem DE, Kokelu AN, et al Pathophysiology and clinical outcomes of pancytopenia in disseminated histoplasmosis: a scoping review. Infection 2025; 53:513–22. 39747737 10.1007/s15010-024-02431-6

[40] Conces DJ Jr, Stockberger SM, Tarver R, Wheat L. Disseminated histoplasmosis in AIDS: findings on chest radiographs. AJR Am J Roentgenol 1993; 160:15–9. 8416614 10.2214/ajr.160.1.8416614

[41] Histoplasma Galactomannan EIA: eurofins; [17 Feb 2026]. Available at: https://www.eurofins-viracor.com/test-menu/30881-30882-33222-33251-histoplasma-galactomannan-eia/#:∼:text=Specificity,cross%2Dreact%20with%20Histoplasma%20antibodies

[42] Rajasingham R, Medina N, Mousquer GT, et al Cost-effectiveness evaluation of routine histoplasmosis screening among people living with advanced HIV disease in Latin America and the Caribbean. PLOS Glob Public Health 2023; 3:e0001861. 37582115 10.1371/journal.pgph.0001861 PMC10427011

[43] World Health Organization . Diagnosing and managing disseminated histoplasmosis among people living with HIV Geneva 2020 [16 Sep 2025]. Available at: https://iris.paho.org/bitstream/handle/10665.2/52304/9789275122495_eng.pdf 36475570

